# Addendum: Early triage of critically ill COVID-19 patients using deep learning

**DOI:** 10.1038/s41467-021-21044-3

**Published:** 2021-02-01

**Authors:** Wenhua Liang, Jianhua Yao, Ailan Chen, Qingquan Lv, Mark Zanin, Jun Liu, SookSan Wong, Yimin Li, Jiatao Lu, Hengrui Liang, Guoqiang Chen, Haiyan Guo, Jun Guo, Rong Zhou, Limin Ou, Niyun Zhou, Hanbo Chen, Fan Yang, Xiao Han, Wenjing Huan, Weimin Tang, Weijie Guan, Zisheng Chen, Yi Zhao, Ling Sang, Yuanda Xu, Wei Wang, Shiyue Li, Ligong Lu, Nuofu Zhang, Nanshan Zhong, Junzhou Huang, Jianxing He

**Affiliations:** 1 China State Key Laboratory of Respiratory Disease and National Clinical Research Center for Respiratory Disease, The First Affiliated Hospital of Guangzhou Medical University, Guangzhou, China; 2 Tencent AI Lab, Shenzhen, China; 3 Hankou Hospital, Wuhan, China; 4 School of Public Health, The University of Hong Kong, Hong Kong SAR, China; 5 Department of Thoracic Surgery, The First Affiliated Hospital of Guangzhou Medical University, Guangzhou, China; 6 Department of Intensive Care Unit, The First Affiliated Hospital of Guangzhou Medical University, Guangzhou, China; 7 Foshan Hospital, Foshan, China; 8 Daye Hospital, Hubei, China; 9 Tencent Healthcare, Shenzhen, China; 10 Department of Respiratory Disease, The Sixth Affiliated Hospital of Guangzhou Medical University, Guangzhou, China; 11 Zhuhai People Hospital, Zhuhai, China

**Keywords:** Predictive medicine, Viral infection, Risk factors

Correction to: *Nature Communications* 10.1038/s41467-020-17280-8, published online 15 July 2020.

The original Article did not reference our previous study^1^, which also presents a clinical prediction model for COVID-19 mortality/critical illness risk. We wish to clarify the similarities and differences between the two studies in terms of cohort populations, methodology and clinical use.

By using the COVID-gram, we can predict the probability of a patient to develop critical illness or death based on the risk score that integrates chest radiographic abnormality, age, hemoptysis, dyspnea, unconsciousness, number of comorbidities, cancer history, neutrophil-to-lymphocyte ratio, lactate dehydrogenase and direct bilirubin. This model was based on a traditional logistic model to predict whether or not a patient will develop into critical illness during the hospital stay.

However, in the real practice, knowing the speed at which the patient will develop critical illness will also influence decision-making regarding treatment. Thus, after finishing the COVID-gram model, we further requested the time information from the data center at China National Health Commission to establish a time-dependent model based on Cox regression. During model selection, we found that deep-learning methods outperformed a traditional linear Cox model. Thus, in our article, we established the deep-learning model to predict critical illness or death at different time points (i.e. 5 days, 10 days, 30 days etc.). In this model, the panel of independent prognostic factors also differs from those used for COVID-gram and includes X-ray abnormalities, age, dyspnea, COPD (chronic obstructive pulmonary disease), number of comorbidities, cancer history, neutrophil/lymphocytes ratio, lactate dehydrogenase, direct bilirubin and creatine kinase. In addition, based on the unique advantages of deep-learning methods through which inherent data relationship can be modelled, we proposed a new imputation scheme to handle missing data automatically, making prediction more widely available in different areas and more robust for errors in data acquisition.

We used a growing dataset managed by the China National Health Commission to build the two models. Both prediction models shared the same dataset for model establishment, which contained 1590 cases from 31 provincial administrative regions across China as of January 31, 2020. For external validation of the COVID-gram, we used 381 cases from Hubei multiple centers until Jan 31, 79 cases from Jinyintan Hopital, Hankou Hospital, Wuhan Union Hospital (Wuhan cohort), 191 cases from Daye Hospital and 73 cases from Foshan Hospital until Feb 28, which did not overlap with the cases from the training cohort. However, in the new investigation for the deep-learning model, in addition to the previous validation dataset (710 cases in total), we collected an updated cohort including previously missing cases from Jinyintan Hopital, Wuhan Union Hospital and especially Hankou Hospital until Feb 28 (*n* = 910; the 79 cases in the previous Wuhan cohort were also included), to generate a new external validation cohort with 1393 cases in total. For the new Wuhan cohort, we added variables not only at hospital admission but also after hospital admission with monitoring data (3–5 time points for each new collected case), and used this data for further tracking and validation. Based on this, we proposed a risk-monitoring model using monitoring data after hospital admission. Furthermore, the external validation cohort was almost doubled in the deep-learning-model paper (increased from 710 to 1393). The deep-learning model showed good performance on the additional validation sets. Collectively, we suggest that users can preferably use the deep-learning model whenever possible, to predict whether a patient will develop critical illness or death at different time points.
