# Supplementary material for: Early triage of critically ill COVID-19 patients using deep learning
Source: Nat Commun. 2020 Jul 15;11:3543. doi: 10.1038/s41467-020-17280-8 (PMC7363899; doi:10.1038/s41467-020-17280-8)
Supplement: Supplementary file 1 — Reporting Summary [file 41467_2020_17280_MOESM1_ESM.pdf]

nature research

Corresponding author(s): DBPR-2020-03-04125

Last updated by author(s): Mar 13, 2020

Reporting Summary

Nature Research wishes to improve the reproducibility of the work that we publish. This form provides structure for consistency and transparency in reporting. For further information on Nature Research policies, see Authors & Referees and the Editorial Policy Checklist .

Statistics

For all statistical analyses, confirm that the following items are present in the figure legend, table legend, main text, or Methods section.

n/a	Confirmed	
☐	☒	The exact sample size (*n*) for each experimental group/condition, given as a discrete number and unit of measurement
☐	☒	A statement on whether measurements were taken from distinct samples or whether the same sample was measured repeatedly
☐	☒	The statistical test(s) used AND whether they are one- or two-sided<br>*Only common tests should be described solely by name; describe more complex techniques in the Methods section.*
☐	☒	A description of all covariates tested
☐	☒	A description of any assumptions or corrections, such as tests of normality and adjustment for multiple comparisons
☐	☒	A full description of the statistical parameters including central tendency (e.g. means) or other basic estimates (e.g. regression coefficient) AND variation (e.g. standard deviation) or associated estimates of uncertainty (e.g. confidence intervals)
☐	☒	For null hypothesis testing, the test statistic (e.g. *F*, *t*, *r*) with confidence intervals, effect sizes, degrees of freedom and *P* value noted<br>*Give P values as exact values whenever suitable.*
☐	☒	For Bayesian analysis, information on the choice of priors and Markov chain Monte Carlo settings
☐	☒	For hierarchical and complex designs, identification of the appropriate level for tests and full reporting of outcomes
☐	☒	Estimates of effect sizes (e.g. Cohen's *d*, Pearson's *r*), indicating how they were calculated

Our web collection on statistics for biologists contains articles on many of the points above.

Software and code

Policy information about availability of computer code

Data collection	No software was used.
Data analysis	the software for data analysis is provided at https://github.com/cojocchen/covid19_critically_ill

For manuscripts utilizing custom algorithms or software that are central to the research but not yet described in published literature, software must be made available to editors/reviewers. We strongly encourage code deposition in a community repository (e.g. GitHub). See the Nature Research guidelines for submitting code & software for further information.

Data

Policy information about availability of data

All manuscripts must include a data availability statement. This statement should provide the following information, where applicable:

- Accession codes, unique identifiers, or web links for publicly available datasets
- A list of figures that have associated raw data
- A description of any restrictions on data availability

The datasets generated during and/or analysed during the current study are not publicly available due to the confidential policy of National Health Commission of China, but are available from the corresponding author Jianxing He upon reasonable request.

Field-specific reporting

Please select the one below that is the best fit for your research. If you are not sure, read the appropriate sections before making your selection.

☒ Life sciences ☐ Behavioural & social sciences ☐ Ecological, evolutionary & environmental sciences

Life sciences study design

All studies must disclose on these points even when the disclosure is negative.

Sample size	the data includes 2983 samples which were all data available to us by the time the manuscript was prepared. It was the largest such kind of data at the time of submission.
Data exclusions	no data were excluded
Replication	External validation based on three cohorts were conducted. The experiments were repeated on separate computers and received consistent results.
Randomization	the primary cohort is randomly divided into training and test sets for evaluation.
Blinding	The data collectors and data analyzers are two groups of investigators and blind to each other.

Reporting for specific materials, systems and methods

We require information from authors about some types of materials, experimental systems and methods used in many studies. Here, indicate whether each material, system or method listed is relevant to your study. If you are not sure if a list item applies to your research, read the appropriate section before selecting a response.

Materials & experimental systems

n/a	Involved in the study
☒	☐ Antibodies
☒	☐ Eukaryotic cell lines
☒	☐ Palaeontology
☒	☐ Animals and other organisms
☐	☒ Human research participants
☒	☐ Clinical data

Methods

n/a	Involved in the study
☒	☐ ChIP-seq
☒	☐ Flow cytometry
☒	☐ MRI-based neuroimaging

Human research participants

Policy information about studies involving human research participants

Population characteristics	the population characteristics is provided in supplementary file (Table 1-5)
Recruitment	We obtained medical records and compiled the data from laboratory-confirmed hospitalized cases with COVID-19 reported to the NHC between November 21st, 2019 and Jan 31st, 2020 for all hospitals listed in appendix.
Ethics oversight	This study was approved by the ethical review committee of the First Affiliated Hospital of Guangzhou Medical University and Hankou hospital, Wuhan.

Note that full information on the approval of the study protocol must also be provided in the manuscript.
