# Supplementary material for: Early triage of critically ill COVID-19 patients using deep learning
Source: Nat Commun. 2020 Jul 15;11:3543. doi: 10.1038/s41467-020-17280-8 (PMC7363899; doi:10.1038/s41467-020-17280-8)
Supplement: Supplementary file 2 — Supplementary information [file 41467_2020_17280_MOESM2_ESM.pdf]

Supplementary Information

Early Triage of Critically-Ill COVID-19 Patients Using Deep Learning

Liang et al.

Supplementary Table 1. Demographic and clinical characteristics of patients with or without critical illness in the training cohort

		Critical illness		
	**Total (n=1590)**	**No (n=1459)**	**Yes (n=131)**	**P Value**
Age (year)	48.9±15.7	47.8±15.2	61.6±14.8	<0.001
Incubation period (day)	5.0±4.1	4.9±4.1	5.7±4.2	0.068
Temperature on admission (℃)	37.3±0.9	37.4±0.9	37.1±0.9	<0.001
Respiratory rate on admission (breath/min)	21.2±12.0	21.1±12.4	23.1±5.9	0.066
Heart rate on admission (beat/min)	88.7±14.6	88.6±14.4	89.7±16	0.383
Systolic pressure on admission (mmHg)	126.1±16.4	125.5±15.6	131.4±22.5	0.005
Diastolic pressure on admission (mmHg)	79.5±25.6	79±11.3	84.7±76.1	0.405
Highest temperature (℃)	38.2±1.6	38.3±1.3	38.1±3.7	0.288
**Sex**				0.017
Male	904/1578 (57.3)	816/1447 (56.4)	88/131 (67.2)	
Female	674/1578 (42.7)	631/1447 (43.6)	43/131 (32.8)	
**Smoking status**				0.004
Never	1479/1590 (93)	1366/1459 (93.6)	113/131 (86.3)	
Former/current	111/1590 (7)	93/1459 (6.4)	18/131 (13.7)	
**Symptoms**				
Degree of Symptom				0.031
0	73/1590 (4.6)	67/1459 (4.6)	6/131 (4.6)	
1	176/1590 (11.1)	170/1459 (11.7)	6/131 (4.6)	
2	353/1590 (22.2)	330/1459 (22.6)	23/131 (17.6)	
3	409/1590 (25.7)	378/1459 (25.9)	31/131 (23.7)	
4	287/1590 (18.1)	258/1459 (17.7)	29/131 (22.1)	
5	158/1590 (9.9)	141/1459 (9.7)	17/131 (13)	

6	76/1590 (4.8)	68/1459 (4.7)	8/131 (6.1)	
7	36/1590 (2.3)	29/1459 (2)	7/131 (5.3)	
8	14/1590 (0.9)	11/1459 (0.8)	3/131 (2.3)	
9	7/1590 (0.4)	6/1459 (0.4)	1/131 (0.8)	
10	1/1590 (0.1)	1/1459 (0.1)	0/131 (0)	
Fever	1351/1536 (88)	1237/1409 (87.8)	114/127 (89.8)	0.572
Conjunctival congestion	10/1345 (0.7)	10/1235 (0.8)	0/110 (0)	1
Nasal congestion	73/1299 (5.6)	64/1191 (5.4)	9/108 (8.3)	0.192
Headache	205/1328 (15.4)	190/1221 (15.6)	15/107 (14)	0.781
Dry cough	1052/1498 (70.2)	959/1372 (69.9)	93/126 (73.8)	0.451
Pharyngalgia	194/1317 (14.7)	181/1207 (15)	13/110 (11.8)	0.482
Productive cough	513/1424 (36)	461/1302 (35.4)	52/122 (42.6)	0.116
Fatigue	584/1365 (42.8)	539/1250 (43.1)	45/115 (39.1)	0.432
Hemoptysis	16/1315 (1.2)	10/1201 (0.8)	6/114 (5.3)	0.001
Dyspnea	331/1394 (23.7)	257/1275 (20.2)	74/119 (62.2)	<0.001
Nausea/vomiting	80/1371 (5.8)	73/1256 (5.8)	7/115 (6.1)	0.84%
Diarrhea	57/1359 (4.2)	52/1244 (4.2)	5/115 (4.3)	0.81
Myalgia/arthralgia	234/1338 (17.5)	215/1229 (17.5)	19/109 (17.4)	1
Chill	163/1333 (12.2)	151/1222 (12.4)	12/111 (10.8)	0.762
**Signs**				
Throat congestion	21/1286 (1.6)	21/1178 (1.8)	0/108 (0)	0.249
Tonsil swelling	31/1376 (2.3)	30/1261 (2.4)	1/115 (0.9)	0.509
Enlargement of lymph nodes	2/1375 (0.1)	1/1261 (0.1)	1/114 (0.9)	0.159
Rash	3/1378 (0.2)	3/1264 (0.2)	0/114 (0)	1
Unconsciousness	20/1421 (1.4)	10/1303 (0.8)	10/118 (8.5)	<0.001
**Comorbidities**				
Any	399/1590 (25.1)	322/1459 (22.1)	77/131 (58.8)	<0.001
No. of comorbidity				<0.001
0	1191/1590 (74.9)	1137/1459 (77.9)	54/131 (41.2)	
1	269/1590 (16.9)	229/1459 (15.7)	40/131 (30.5)	
2	88/1590 (5.5)	68/1459 (4.7)	20/131 (15.3)	
3	34/1590 (2.1)	20/1459 (1.4)	14/131 (10.7)	
4	5/1590 (0.3)	4/1459 (0.3)	1/131 (0.8)	
5	3/1590 (0.2)	1/1459 (0.1)	2/131 (1.5)	
COPD	24/1590 (1.5)	12/1459 (0.8)	12/131 (9.2)	<0.001

Diabetes	130/1590 (8.2)	99/1459 (6.8)	31/131 (23.7)	<0.001
Hypertension	269/1590 (16.9)	216/1459 (14.8)	53/131 (40.5)	<0.001
Cardiovascular disease	59/1590 (3.7)	46/1459 (3.2)	13/131 (9.9)	0.001
Cerebrovascular disease	30/1590 (1.9)	20/1459 (1.4)	10/131 (7.6)	<0.001
Hepatitis B infection	28/1590 (1.8)	25/1459 (1.7)	3/131 (2.3)	0.498
Cancer History	18/1590 (1.1)	11/1459 (0.8)	7/131 (5.3)	<0.001
Chronic kidney disease	21/1590 (1.3)	15/1459 (1)	6/131 (4.6)	0.005
Immunodeficiency	3/1590 (0.2)	2/1459 (0.1)	1/131 (0.8)	0.227
**Abnormal chest images**				
Radiograph	243/1590 (15.3)	184/1459 (12.6)	59/131 (45)	<0.001
Degree of abnormality				<0.001
0	1347/1590 (84.7)	1275/1459 (87.4)	72/131 (55)	
1	138/1590 (8.7)	104/1459 (7.1)	34/131 (26)	
2	69/1590 (4.3)	59/1459 (4)	10/131 (7.6)	
3	36/1590 (2.3)	21/1459 (1.4)	15/131 (11.5)	
Computed tomography	1130/1590 (71.1)	1035/1459 (70.9)	95/131 (72.5)	0.763
Degree of abnormality				0.016
0	460/1590 (28.9)	424/1459 (29.1)	36/131 (27.5)	
1	492/1590 (30.9)	466/1459 (31.9)	26/131 (19.8)	
2	291/1590 (18.3)	258/1459 (17.7)	33/131 (25.2)	
3	248/1590 (15.6)	224/1459 (15.4)	24/131 (18.3)	
4	99/1590 (6.2)	87/1459 (6)	12/131 (9.2)	
**Hubei**				<0.001
Yes	647/1590 (40.7)	552/1459 (37.8)	95/131 (72.5)	
No	943/1590 (59.3)	907/1459 (62.2)	36/131 (27.5)	
**Wuhan-contacted**				0.004
Yes	1334/1590 (83.9)	1213/1459 (83.1)	121/131 (92.4)	
No	256/1590 (16.1)	246/1459 (16.9)	10/131 (7.6)	

Data are mean±standard deviation, n/N (%), where N is the total number of patients with available data. P values are calculated by χ^2 test (two-sided), Fisher's exact test, or Mann-Whitney U test. COPD=chronic obstructive pulmonary disease. (highlighted lines are features selected by our model).

Supplementary Table 2. Laboratory findings of patients with or without critical illness on admission to hospital in the training cohort.

		**Critical illness**		
	**Total (n=1590)**	**No (n=1459)**	**Yes (n=131)**	**P Value**
Total urine volume, ml per day	794.6±901.6	622.1±855.4	1155.1±907.8	0.021
PaO2 (with oxygen inhalation), mmHg	84.3±36.2	86.5±36	67.2±33.3	<0.001
FiO2, %	27.5±16.6	26.6±14.7	33.2±25.2	0.116
PaO2 (without oxygen inhalation), mmHg	92.7±13.5	93.6±12.5	85.8±18.2	0.002
White blood cell count, × 10^9 per L	5.5±3	5.3±2.6	8.5±4.8	<0.001
Lymphocyte count, × 10^9 per L	1.4±3.1	1.5±3.3	0.7±0.4	<0.001
Platelet count, × 10^9 per L	179.5±70.7	180.1±70.4	173.4±73.7	0.368
Hemoglobin, g/L	123.5±43.9	124.2±43.9	115.5±43.5	0.058
CD4+ T lymphocyte count, × 10^9 per L	157.8±279.8	169.3±299	100.2±142.4	0.129
CD8+ T lymphocyte count, × 10^9 per L	82.3±141.7	88.6±152.7	51.1±58.6	0.075
CD4/CD8 ratio	2±1	2±0.9	2±1.6	0.949
C-reactive protein, mg/L	34.8±49.2	30.6±43.8	84.5±76.3	<0.001
Procalcitonin, ng/mL	0.7±9.8	0.8±10.3	0.6±1.4	0.879
Lactate dehydrogenase, U/L	314.3±693.7	273.6±135.2	723.6±2239.5	0.071
Aspartate aminotransferase, U/L	49.7±451.9	34.1±20.9	205.1±1493.4	0.272
Alanine aminotransferase, U/L	43.1±242.5	34.4±37.2	130.1±795.9	0.257
Direct bilirubin, mmol/L	4±2.7	3.7±2.3	6.5±4.1	<0.001
Indirect bilirubin, mmol/L	7.4±4.5	7.2±4.3	8.9±5.3	0.007
Total bilirubin, mmol/L	11.8±14.2	11.4±14.7	15.2±8.4	0.016
Creatine kinase, U/L	135.5±246.7	123±125.3	258.9±702.8	0.082
Creatinine, µmol/L	76±71.4	71.8±54.2	118.7±158.4	0.006
Hypersensitive troponin I, pg/mL	76.3±586.4	42.7±439	288.1±1124.2	0.097
Albumin, g/L	38.7±9.1	39.3±8.9	32.6±8.9	<0.001
Sodium, mmol/L	140.5±50.4	139.7±41.2	148.7±103.1	0.398
Potassium, mmol/L	4.4±6.4	4.4±6.7	4.1±0.8	0.625

Chlorine, mmol/L	103.8±28.2	103.7±29.5	105±4.8	0.685
D-dimer, mg/L	25.5±138.2	26.3±144.8	19.1±70.1	0.636
Prothrombin time, s	17.4±48.2	17.6±50.2	15.9±24.1	0.762
Activated partial thromboplastin time, s	42.5±143.7	43.3±150.6	34.8±50.9	0.597
Neutrophil-lymphocyte ratio	5.1±5.6	4.4±3.8	12.7±12.4	<0.001

Data are mean±standard deviation. P values are calculated by χ^2 test (two-sided), Fisher's exact test, or Mann-Whitney U test. (highlighted lines are features selected by our model).

Supplementary Table 3. Selected clinical features for patients in external validation cohort I (Wuhan cohort).

	Critical illness			
	**Total (n=940)**	**No (n=846)**	**Yes (n=94)**	**P Value**
Age (year)	56.0±16.0	54.3±15.5	70.8±12.0	<0.001
Cancer History	30/751 (4.0)	25/667 (3.7)	5/84 (6.0)	0.368
Dyspnea	328/751 (43.7)	272/667 (40.8)	56/84 (66.7)	<0.001
COPD	21/749 (2.8)	15/665 (2.3)	6/84 (7.1)	0.023
No. of comorbidity	0.6±0.9	0.6±0.9	1.0±1.0	<0.001
Radiograph	940/940 (100)	846/846 (100)	94/94 (100)	1
Lactate dehydrogenase, U/L	292.1±192.8	261.2±122.4	544.2±383.1	<0.001
Direct bilirubin, mmol/L	4.5±12.6	3.6±2.6	12.7±37.1	<0.001
Creatine kinase, U/L	122.4±168.6	108.3±153.0	237.2±233.2	<0.001
Neutrophil-lymphocyte ratio	7.1±9.2	5.9±7.5	18.1±13.6	<0.001

Data are mean±standard deviation. P values are calculated by χ^2 test, Fisher's exact test, or Mann-Whitney U test.

Supplementary Table 4. Selected clinical features for patients in external validation cohort II (Hubei cohort).

	Critical illness			
	**Total (n=380)**	**No (n=371)**	**Yes (n=9)**	**P Value**
Age (year)	39.3±96.1	38.7±97.2	66.5±11.1	<0.001
Cancer History	2/55 (3.6)	2/50 (4.0)	0/5 (0.0)	1
Dyspnea	43/294 (14.6)	39/285 (13.7)	4/9 (44.4)	0.029
COPD	4/55 (7.3)	4/50 (8.0)	0/5 (0.0)	1
No. of comorbidity	0.3±0.6	0.3±0.6	1.4±1.1	<0.001
Radiograph	29/380 (7.6)	26/371 (7.0)	3/9 (33.3)	0.025
Lactate dehydrogenase, U/L	250.2±114.0	240.1±83.6	607.5±306.9	<0.001
Direct bilirubin, mmol/L	4.2±5.5	4.2±5.5	4.3±3.7	0.281
Creatine kinase, U/L	148.6±223.0	149.7±225.7	107.6±70.8	0.466
Neutrophil-lymphocyte ratio	5.5±7.2	5.0±4.1	22.4±31.6	0.002

Data are mean±standard deviation. P values are calculated by χ^2 test (two-sided), Fisher's exact test, or Mann-Whitney U test.

Supplementary Table 5. Selected clinical features for patients in external validation cohort III (Guangdong cohort).

		Critical illness		
	**Total (n=73)**	**No (n=70)**	**Yes (n=3)**	**P Value**
Age (year)	43.8±15.4	43.0±14.8	71.0±5.0	0.015
Cancer History	0/73 (0.0)	0/71 (0.0)	0/2 (0.0)	1
Dyspnea	18/73 (24.7)	16/71 (22.5)	2/2 (100.0)	0.058
COPD	0/73 (0.0)	0/71 (0.0)	0/2 (0.0)	1
No. of comorbidity	0.3±0.7	0.3±0.6	2.0±1.0	0.003
Radiograph	61/73 (83.6)	59/71 (83.1)	2/2 (100.0)	1
Lactate dehydrogenase, U/L	466.6±189.5	463.6±188.1	571.5±208.5	0.277
Direct bilirubin, mmol/L	2.6±3.6	2.5±3.6	4.0±2.3	0.184
Creatine kinase, U/L	60.8±42.1	57.8±38.1	168.5±31.5	0.013
Neutrophil-lymphocyte ratio	4.0±6.1	3.2±1.8	32.8±19.4	0.009

Data are mean±standard deviation. P values are calculated by χ^2 test (two-sided), Fisher's exact test, or Mann-Whitney U test.

Supplementary Table 6. Optimized hyper-parameters of the Deep-Learning Survival Cox model.

Hyper-parameters	**Optimal values**
Hidden layer 1 size	27
Hidden layer 2 size	12
Learning rate	0.00128
Learning rate decay	0.99
Dropout rate	0.2178
Training epochs	1400

Supplementary Table 7. Comparison between our deep learning survival Cox model and the classic Cox model

	**C-Index**		**AUROC**		**p-AUROC**	
	**Classic Cox**	**Deep Learning (p-value)**	**Classic Cox**	**Deep Learning (p-value)**	**Classic Cox**	**Deep Learning (p-value)**
Internal validation	0.876	0.894 (0.025)	0.889	0.911 (0.09)	0.809	0.861 (0.01)
Wuhan cohort	0.857	0.870 (<0.001)	0.863	0.881 (0.22)	0.773	0.812 (0.10)
Hubei cohort	0.765	0.769 (0.472)	0.813	0.819 (0.50)	0.598	0.596 (0.87)
Guangdong cohort	0.958	0.967 (0.032)	0.957	0.967 (0.25)	0.937	0.944 (0.32)

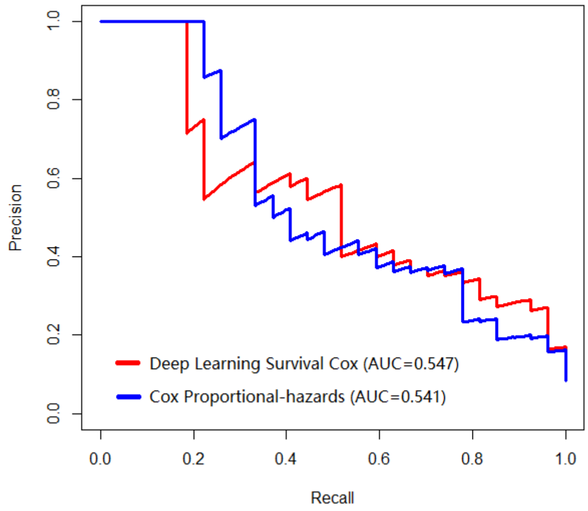

Supplementary Figure 1. Precision-recall curves for the internal validation set.

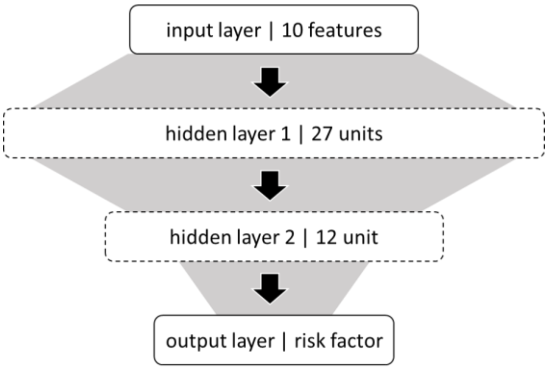

Supplementary Figure 2. Illustration of the feed forward neural network architecture of the proposed deep survival model.

Supplementary Note

List of hospitals reporting cases

Wuhan Jinyintan hospital, Union Hospital Affiliated to Tongji Medical College of Huazhong University of science and technology, Wuhan Central Hospital, Wuhan first hospital, Chengdu Public Health Clinical Medical Center, Huangshi Central Hospital, Shenzhen Third People's Hospital, Wuhan Pulmonary Hospital, Tianyou Hospital Affiliated to Wuhan University of science and technology, Changsha First Hospital, The third people's Hospital of Hainan Province, Huanggang Central Hospital, Wenling first people's Hospital, Yichang Third People's Hospital, Taihe Hospital Affiliated to Hubei Medical College, Xiantao first people's Hospital, Wuhan Huangpi District People's Hospital, Jingzhou Chest Hospital, Jingzhou first people's Hospital, Shanghai Public Health Clinical Center, Beijing You'an Hospital Affiliated to Capital Medical University, Zhengzhou Sixth People's Hospital, Chongqing Three Gorges Central Hospital, The ninth Affiliated Hospital of Guangxi Medical University, Hangzhou Xixi hospital, Nanjing Second Hospital, Suzhou Fifth People's Hospital, The first hospital of Zhejiang Province, The Fifth Affiliated Hospital of Zhongshan University, Huangshi traditional Chinese medicine hospital, Yangjiang people's Hospital, Zhongxian hospital, the First Affiliated Hospital of Chongqing Medical University, Anqing Municipal Hospital, Changzhou Third People's Hospital, Guangzhou first people's Hospital, Harbin infectious diseases hospital, Tianmen first people's Hospital, Wuxi People's Hospital, Wuhan fifth hospital, Xishuangbanna Dai Autonomous Prefecture People's Hospital, Chongqing Iron and Steel General Hospital, Daye people's Hospital, Nanxishan Hospital of Guangxi Zhuang Autonomous Region, Jiaxing First Hospital, Jiangling people's Hospital, Jinzhong infectious disease hospital, Lanzhou Pulmonary Hospital, Liuzhou people's Hospital, Ma'anshan He county people's Hospital, The First Affiliated Hospital of Nanchang University, Ningbo Yinzhou people's hospital medical community, Shaoxing people's Hospital, Shijiazhuang fifth hospital, Taizhou Enze Medical Center, Xinyang Central Hospital, Yueyang No.1 People's Hospital, Zhanjiang Central People's Hospital, The First Affiliated Hospital of Zhengzhou University, Shenzhen Hospital of Chinese Academy of Sciences, Chongqing Kaizhou District People's Hospital, Chongqing Changshou District People's Hospital, Chongqing Yunyang County People's Hospital, Ankang Central Hospital, Chenzhou Second People's Hospital, Datong Fourth People's Hospital, Dengzhou people's Hospital, Fengjie people's Hospital, Foshan first people's Hospital, Fuyang Second People's Hospital, Gongyi people's Hospital, Guangshan people's Hospital, Guoyao Dongfeng General Hospital, Hainan people's Hospital, The Second Affiliated Hospital of Hainan Medical College, The first people's Hospital of Xiaoshan District, Hangzhou, Huaihua first people's Hospital, Jiashan first people's Hospital, Lu'an people's Hospital, Affiliated Hospital of Qingdao University, Qingyuan people's Hospital, Quanzhou County People's Hospital, Rizhao people's Hospital, Shaodong people's Hospital, Shiyan Xiyuan Hospital, Tongling people's Hospital, Wenzhou People's Hospital, Wenzhou Central Hospital, The Second Affiliated Hospital of Wenzhou Medical University, Wuxi Fifth People's Hospital, Wuhan Youfu hospital, Xi'an eighth hospital, Xinxiang infectious disease hospital, Yangxin County People's Hospital, Yuebei Second People's Hospital, Yunnan infectious diseases hospital, Zhaoqing first people's Hospital, Zhaozhou County People's Hospital, Shao Yifu Hospital Affiliated to Zhejiang University School of Medicine, Zhijiang people's Hospital, People's Hospital

of Dianjiang County, Chongqing, Chongqing Jiulongpo first people's Hospital, Chongqing Shizhu Tujia Autonomous County People's Hospital, The first people's Hospital of Wanzhou District, Chongqing, Yongchuan Hospital Affiliated to Chongqing Medical University, Anguo hospital, The Third Hospital of Peking University, peking university shenzhen hospital , BOLUO people's Hospital, Changde Lixian people's Hospital, Changde Second People's Hospital, Chenzhou Central Hospital, Chengjiang people's Hospital, Dalian Central Hospital, Danzhou people's Hospital, Dengzhou Central Hospital, Feidong County People's Hospital, Fuzhou Nanfeng County Hospital, Ganzhou Fifth People's Hospital, Gao'an people's Hospital, Public Security County People's Hospital, Affiliated Hospital of Guangdong Medical University, The Sixth Affiliated Hospital of Guangzhou Medical University, Affiliated Hospital of Guizhou Medical University, Hangzhou first people's Hospital, Hangzhou Lin'an District People's Hospital, Nanpi County Hospital of traditional Chinese medicine of Hebei Province, Henan people's Hospital, Hefeng County Central Hospital, Hohhot First Hospital, Huludao Central Hospital, The First Affiliated Hospital of Hunan Medical College, Shenzhen Union Hospital of Huazhong University of science and technology, Huaibei people's Hospital, Huangshi Second Hospital, Huangchuan people's Hospital, Huizhou Zhongda Huiya hospital, Huizhou Central People's Hospital, Jining first people's Hospital, Jianshi County People's Hospital, Fengcheng people's Hospital of Jiangxi Province, Jiangyou infectious diseases hospital, Jieyang people's Hospital, Jinhua Central Hospital, Jinzhong Pingyao people's Hospital, Jingjiang people's Hospital, The Second Affiliated Hospital of Kunming Medical University, Laifeng County Central Hospital, Yueqing people's Hospital, Lijiang people's Hospital, Lixin people's Hospital, The Fourth People's Hospital of Lianyungang, Linqu County People's Hospital, Linyi people's Hospital, Longxi first people's Hospital, Min Da Hospital, Minqing County General Hospital, Nantong Third People's Hospital, Nanyang Central Hospital, The First Affiliated Hospital of Nanyang Medical College, Nanyang Oilfield General Hospital, Ningbo First Hospital, The Fourth People's Hospital of Ningxia, Pingxiang Second People's Hospital, Quzhou Kecheng District People's Hospital, Qujing maternal and child hospital, Ruian people's Hospital, The First Affiliated Hospital of Xiamen University, Shangcheng County People's Hospital, Shanghai Baoshan Dachang hospital, Shanghai Pudong New Area Gongli Hospital , People's Hospital of Yushan County, Jiangxi Province, Shangrao City, Xuanwu Hospital of Capital Medical University, Sichuan Mianyang 404 hospital, Sixian Hospital of traditional Chinese Medicine, Suihua First Hospital, Suiping County People's Hospital, Tianjin Fourth Central Hospital, Tianjin Haihe hospital, Tiantai County People's Hospital, Tongchuan Mining Bureau Central Hospital, Tongren people's Hospital, Weihai Central Hospital, The First Affiliated Hospital of Wenzhou Medical University, Wuzhou Third People's Hospital, Armed police Hubei provincial general team hospital, Xixian people's Hospital, Longshan County People's Hospital in Western Hunan, Xiangcheng first people's Hospital, The Sixth People's Hospital of Xinjiang Uygur Autonomous Region, The First Affiliated Hospital of Xinjiang Medical University, Xinmi Hospital of traditional Chinese Medicine, Xinxiang County People's Hospital, Xinye people's Hospital, Xinyang first people's Hospital, Xinyang Hospital of traditional Chinese Medicine, Xuanen County People's Hospital, Xinhua Hospital, Yili Prefecture, Yongzhou Central Hospital, Yuyao people's Hospital, Changchun infectious disease diagnosis and treatment center, Changsha eighth hospital, Changsha first people's Hospital, 921st Hospital of the joint service support force of the Chinese people's Liberation Army, Central theater General Hospital of the Chinese people's Liberation Army, The First Affiliated Hospital of China Medical University, The Third Affiliated Hospital of Zhongshan University, Zhongshan Second People's Hospital,

Chongqing Chengkou people's Hospital, Chongqing Hechuan District People's Hospital, Chongqing Red Cross Hospital, Zhoushan women's and children's Hospital, Zhoukou infectious diseases hospital, Zhuzhou first people's Hospital, Zhumadian Central Hospital, Anlong people's Hospital, Anxi County Hospital, Anyang Fifth People's Hospital, Anyang People's Hospital, Anyuan people's Hospital, Badong County Ethnic hospital, Wuyuan County People's Hospital of Bayannur City, Baise people's Hospital, The First Affiliated Hospital of Bengbu Medical College, Baoding first Central Hospital, Changping District Hospital of Beijing Municipality, Changping District Hospital of traditional Chinese and Western medicine of Beijing, Beijing Chuiyangliu Hospital , Beijing Center for Disease Control and Prevention, Mentougou District Hospital of Beijing Municipality, Shunyi District Hospital of Beijing Municipality, Beijing Xicheng District Guangwai hospital, Oriental Hospital of Beijing University of traditional Chinese Medicine, Benxi Sixth People's Hospital, Binzhou Central Hospital, Bozhou people's Hospital, Cangnan Third People's Hospital, People's Hospital of Anxiang County, Changde City, Changde first people's Hospital, Chaoyang Second Hospital, Chengdu Handan people's Hospital, Chengde Third Hospital, Chizhou people's Hospital, Chongxin County People's Hospital, Chongyi people's Hospital, Affiliated Hospital of North Sichuan Medical College, Dazhou Central Hospital, Dali first people's Hospital, The Second Affiliated Hospital of Dalian Medical University, The First Affiliated Hospital of Dalian Medical University, Danyang people's Hospital, Daocheng people's Hospital, Deqing people's Hospital, Dezhou Second People's Hospital, Dezhou people's Hospital, Dezhou Qingyun people's Hospital, Dingyuan County General Hospital, Dongfang people's Hospital, Dongguan Ninth People's Hospital, Dongguan Nancheng hospital, Dongyang people's Hospital, Enshi Central Hospital, Erlianhot hospital, Fangchenggang first people's Hospital, Nanzhuang hospital, Chancheng District, Foshan City, Foshan Nanhai District Third People's Hospital, Lishui hospital, Nanhai District, Foshan City, The First Affiliated Hospital of Fujian Medical University, Zhangzhou Hospital Affiliated to Fujian Medical University, Fuzhou Changle district hospital, Fuzhou Anle County Hospital, Fuzhou Fifth People's Hospital, Fuzhou Dongxiang District People's Hospital, Fuyang District First People's Hospital, Ganzhou Longnan County People's Hospital, Gaolan County People's Hospital, Gongcheng Yao Autonomous County People's Hospital, Gushi people's Hospital, Guang'an people's Hospital, Guangdong Hospital of traditional Chinese Medicine, Guangzhou Eighth People's Hospital, Guangzhou 12th people's Hospital, Shenzhen Hospital of Guangzhou University of traditional Chinese Medicine, People's Hospital of Guiding County, Hanjiang Hospital of Sinopharm, Harbin Acheng District People's Hospital, Nangang District People's Hospital of Harbin, The First Affiliated Hospital of Harbin Medical University, Haikou People's Hospital, Hainan West Central Hospital, Handan Sixth Hospital, Handan Central Hospital, Hanshan people's Hospital, Hangzhou Dingqiao hospital, The third people's Hospital of Yuhang District, Hangzhou, The first people's Hospital of Yuhang District, Hangzhou, Minzhou people's Hospital, Hefei Sixth People's Hospital (Hefei infectious diseases hospital), He Xian Memorial Hospital, Hebei Chest Hospital, Hechi people's Hospital, Hejin people's Hospital, The First Affiliated Hospital of Henan University of science and technology, Zhangye people's Hospital Affiliated to Hexi University, Heyuan people's Hospital, Heze Municipal Hospital, Heilongjiang provincial hospital , South Yunnan Central Hospital of Honghe Prefecture, Hulunbuir Manzhouli hospital, Hunan Youxian people's Hospital, The First Affiliated Hospital of Hunan University of traditional Chinese Medicine, China Resources WISCO General Hospital, Huaihua Chenxi County People's Hospital, Huai'an Fourth People's Hospital, Huainan Mashan infectious disease hospital, Huangshan people's Hospital,

Huangshi fifth hospital, Huichang people's Hospital, Huining County People's Hospital, Huizhou first people's Hospital, Jixi people's Hospital, Qianan County People's Hospital of Jilin Province, Jinan Fourth People's Hospital, Jining second people's Hospital, Affiliated Hospital of Jining Medical College, Shunde Hospital Affiliated to Jinan University, Jiamusi Fujin Central Hospital, Huachuan County People's Hospital of Jiamusi, Jiahe County People's Hospital, Jianshi County Hospital of traditional Chinese Medicine, Jiangshan people's Hospital, People's Hospital of Le'an County, Jiangxi Province, Jiangxi Provincial People's Hospital, Jinxian County People's Hospital, Jingmen Chest Hospital, Kunming Second People's Hospital, Laixi people's Hospital, The second hospital of Lanzhou University, Lancang Second People's Hospital, Leping people's Hospital, Leshan people's Hospital, Lengshuijiang people's Hospital, Lianjiang county hospital, The first people's Hospital of Lianyungang, Liaoning Chaoyang Disease Control Center Hospital, Liaocheng people's Hospital, Linshui people's Hospital, Linhai Second People's Hospital, Linxia people's Hospital, Linyi Lanshan District People's Hospital, Linying County People's Hospital, Liuyang people's Hospital, Loudi first people's Hospital, Loudi Central Hospital, Luzhou people's Hospital, Lushan County People's Hospital, The Third Affiliated Hospital of Army Medical University, The First Affiliated Hospital of Army Medical University, Luoping County People's hospital official website, Luoyuan County Hospital, Luohe Sixth People's Hospital, MAANSHAN Fourth People's Hospital, MAANSHAN Hospital of traditional Chinese Medicine, Coal Industry General Hospital, The first people's Hospital of Mengcheng County, Mianyang people's Hospital, People's Hospital of Mianchi County, Mudanjiang Second People's Hospital, The Second Affiliated Hospital of Nanchang University, Fuzhou Fifth Hospital Affiliated to Fuzhou Medical College of Nanchang University, Nanchong Central Hospital, Southern Hospital of Southern Medical University, The First Affiliated Hospital of Nanhua University, Public Health Hospital of Nanhua University, Nanhua Hospital Affiliated to Nanhua University, Xiangtan Hospital Affiliated to Nanhua University, Nanning Fourth People's Hospital, People's Hospital of Neihuang County, Ningbo Second Hospital, Community health service center, Baihe street, Jiangdong District, Ningbo City, Li Huili Hospital of Ningbo Medical Center, Mindong Hospital of Ningde City, Ningde Xiapu County Hospital, Ningdu County People's Hospital, Ningguo people's Hospital, People's Hospital of Ninglang Yi Autonomous County, Ouhai District Third People's Hospital, Pingdingshan infectious diseases hospital, Pingguo people's Hospital, Qiqihar seventh hospital, The First Affiliated Hospital of Qiqihar Medical College, Qidong Third People's Hospital, Qingdao Chengyang people's Hospital, Qingdao Huangdao District People's Hospital, The Fourth People's Hospital of Qinghai Province, Qingyuan people's Hospital, Quanjiao people's Hospital, Luojiang District Hospital of Quanzhou City, Queshan County People's Hospital, Renshou people's Hospital, Wulian people's Hospital of Rizhao, Rongcheng people's Hospital, Runan people's Hospital, Rushan people's Hospital, Sanming integrated hospital of traditional Chinese and Western Medicine, Zhongshan Hospital Affiliated to Xiamen University, Shandong Provincial Hospital , Shandong Chest Hospital, Shanxi Bethune hospital, Ruicheng County People's Hospital of Shanxi Province, The second hospital of Yuncheng City, Shanxi Province, The First Affiliated Hospital of Shantou University Medical College, Shantou Central Hospital, Shangluo Luonan people's Hospital, The Second Affiliated Hospital of Fudan, Shanghai, Renji Hospital Affiliated to Shanghai Jiaotong University School of Medicine, Ruijin Hospital Affiliated to Shanghai Jiaotong University School of Medicine, Shanghai 10th people's Hospital, Shanghai Fengxian District Central Hospital, Anting hospital, Jiading District, Shanghai, Shanghai Tongren Hospital, Shangrao Guangxin District

People's Hospital, Shangrao people's Hospital, Shangrao Wannian County People's Hospital, Shangrao Yongxiu people's Hospital, Shenzhen Bao'an District Hospital of traditional Chinese Medicine, Shenzhen Sixth People's Hospital, Shenzhen Longgang District People's Hospital, Shenzhen Pingshan people's Hospital, Shenyang hospital, Shiyan people's Hospital, Shiyan integrated traditional Chinese and Western Medicine Hospital, The First Affiliated Hospital of Shihezi University Medical College, Shishi General Hospital, Shishou people's Hospital, Shuangfeng County People's Hospital, West China Hospital of Sichuan University, Sihui people's Hospital, Songzi people's Hospital, Suichuan County People's Hospital, Taizhou Second People's Hospital, Taizhou first people's Hospital, Taiyuan Fourth People's Hospital, Tanghe County People's Hospital, Tianjin Third Central Hospital, Tianjin Fifth Central Hospital, Tianjin First Central Hospital, Tianjin People's Hospital, Tianjin Xiqing hospital, Tianjin Medical University General Hospital , tianjin hospital, Tianquan Tianyuan hospital, Tieling Central Hospital, Tonghua people's Hospital, Tongbai County Central Hospital, Wanning people's Hospital, Weixin County People's Hospital, Weishan people's Hospital, Wenzhou Longwan District First People's Hospital, Wenzhou Yongjia hospital, Wenzhou Hospital of traditional Chinese Medicine, People's Hospital of Woyang County, Wuwei people's Hospital, Wuxi Fourth People's Hospital, Wuzhong people's Hospital, Wuhan Caidian District People's Hospital, People's Hospital of Wuhan University, Wuhan Pulmonary Hospital, Caidian District People's Hospital of Wuhan, Wuhan Sixth Hospital, Wuhan Dongxihu District People's Hospital, Wuhan commercial staff hospital, Wuhan Wuchang hospital, Wuhan hospital of traditional Chinese Medicine, Wuyang County People's Hospital, The First Affiliated Hospital of Xi'an Jiaotong University, Central Hospital of Xi'an Mining Bureau, Xiping County People's Hospital, Xishuangbanna mental health center, Xilingol League hospital, Xilinhot, Xianyang Qianxian people's Hospital, Xianyang Central Hospital, Xianyang Wugong County People's Hospital, Xiangtan Xiangtan County People's Hospital, Xinhuang people's Hospital, The seventh division hospital of Xinjiang production and Construction Corps, Xinyu people's Hospital, Xinyang Third People's Hospital, Xinyang Fifth People's Hospital, Suqian infectious disease control center, Affiliated Hospital of Xuzhou Medical University, Xuyong County People's Hospital, Haiyang people's Hospital of Yantai, Yantai Qishan hospital, Yan'an University Affiliated Hospital, Yancheng Dafeng people's Hospital, Yan Fen clinic, Yangzhou Third People's Hospital, Yangquan Third People's Hospital, Yangshuo people's Hospital, Yichang first people's Hospital, Yichang Central People's Hospital, People's Hospital of Zigui County, Yichang, Yimen people's Hospital, Yiyang Anhua people's Hospital, South County People's Hospital of Yiyang, People's Hospital of Datong Lake District, Yiyang City, Yinchuan first people's Hospital, Yingkou Third People's Hospital, Yingcheng people's Hospital, Yongfu people's Hospital, Yongjia people's Hospital, Yongtai County Hospital, Yuzhong first people's Hospital, Yulin Red Cross Hospital, People's Hospital of Jiangchuan District, Yuxi City, Yuxi people's Hospital, Yueyang Second People's Hospital, The third people's Hospital of Yunnan Province, Affiliated Hospital of Changchun Beihua University, Changjiang Shipping General Hospital, Changzhi Changzhi people's Hospital, Zhao'an County Hospital, The First Affiliated Hospital of Zhejiang University Medical College, Taizhou Hospital of Zhejiang Province, Zhenjiang first people's Hospital, Zhengzhou Central Hospital, Ningbo Huamei Hospital of Chinese Academy of Sciences, PLA Navy General Hospital, 985th Hospital of the joint service support force of the Chinese people's Liberation Army, 924th Hospital of the joint service support force of the Chinese people's Liberation Army, Aviation General Hospital of China Medical University, Xiangya Third Hospital of Central South University, Huiya hospital,

the First Affiliated Hospital of Zhongshan University, Zhongwei people's Hospital, Zhongxiang people's Hospital, Chongqing Liangjiang New Area first people's Hospital, Chongqing Bishan District People's Hospital, Fengdu County People's Hospital of Chongqing, Chongqing Fuling Central Hospital, Chongqing public health medical treatment center, Chongqing Liangping District People's Hospital, Chongqing Tongnan District People's Hospital, Chongqing Wanzhou District Shanghai hospital, Central Hospital of Wuling Town, Wanzhou District, Chongqing, Chongqing Xiushan people's Hospital, The Third Affiliated Hospital of Chongqing Medical University, The First Affiliated Hospital of Chongqing Medical University, Zhoukou Central Hospital, Zhuhai People's Hospital, Zibo Central Hospital, Zigong Rongxian people's Hospital, Zigong first people's Hospital, Zhuhai integrated traditional Chinese and Western Medicine Hospital, Tianhe District Center for Disease Control and Prevention, Shanwei people's Hospital, Shantou Chenghai District People's Hospital, The Second Affiliated Hospital of Shantou University Medical College, Shantou Chaonan Minsheng hospital, Luocun hospital, Nanhai District, Luhe County People's Hospital, The first naval hospital of the southern theater of the PLA, Jianghai work station of Jiangmen disease control and Prevention Center, Huizhou Third People's Hospital, Huadu District People's Hospital, The Third Affiliated Hospital of Guangzhou Medical University, Guangzhou Chest Hospital, Guangzhou Haizhu Center for Disease Control and Prevention, Guangning people's Hospital, Zhuhai Hospital of Guangdong Hospital of traditional Chinese Medicine, Guangdong hydropower hospital Co. Ltd, Guangdong Provincial People's Hospital, The second people's Hospital of Guangdong Province, Panyu District Central Hospital, Dongguan Dalang hospital, Chaozhou Central Hospital
